# Prepublication abstract-only reports compared with full-text manuscripts for randomised controlled trials in inflammatory bowel disease: a systematic review

**DOI:** 10.1136/bmjgast-2023-001334

**Published:** 2024-03-07

**Authors:** Vassiliki Sinopoulou, Morris Gordon, Gordon William Moran, Abdullah Mohammed Abousaleh ma Egiz, Sanjana Phlananthachai, Aditi Rane, Ahmed Hussein Ali Al-Tameemi

**Affiliations:** 1 University of Central Lancashire, Preston, UK; 2 BEST Unit, University of Central Lancashire, Preston, UK; 3 Nottingham University Hospitals NHS Trust - City Campus, Nottingham, UK

**Keywords:** IBD, META-ANALYSIS, STATISTICS

## Abstract

**Introduction:**

Randomised controlled trials (RCTs) of key therapies in inflammatory bowel disease (IBD) are often presented and available as abstracts for significant periods of time prior to full publication, often being employed to make strategic and clinical prescribing decisions. We compared the concordance of prepublication abstract-only reports and their respective full-text manuscripts.

**Methods:**

Pairs of full-text manuscripts and their respective prepublication abstract-only reports for the same RCT outcomes, at the same time point of analysis were included. The RCTs were on treatments for IBD with full-text manuscripts published between 2010 and 2023.

**Results:**

We found 77 pairs of full-text manuscripts and their prepublication abstract-only reports. There were significant mismatches in the reporting of stated planned outcomes (65/77 matched, p<0.001) and primary outcomes reported in their results sections (67/77, p<0.001); trial registrations (34/65, p<0.001); the number of randomised participants (49/77, p=0.18); participants reaching end of study (21/71, p<0.001) and primary outcome data (40/73, p<0.001). Authors conclusions matched (75/77, p=0.157). Authors did not provide explicit or implied justifications for the absence or non-concordance for any of the above items.

**Conclusions:**

Abstract-only reports have consistent issues with both limited reporting of key information and significant differences in data when compared with their later full-text publications. These are not related to further recruitment of patients or word count limitations and are never explained. As abstracts are often used in guidelines, reviews and stakeholder decision-making on prescribing, caution in their use is strongly suggested. Further work is needed to enhance minimum reporting standards in abstract-only works and ensure consistency with final published papers.

**WHAT IS ALREADY KNOWN ON THIS TOPIC**

- Data from abstract-only reports of randomised controlled trials are often used as evidence sources for clinical and strategic decision-making in inflammatory bowel disease. It is not known whether they are up to par for this purpose.

**WHAT THIS STUDY ADDS**

- Our findings suggest that abstract-only reports are often inconsistent in their reporting, compared with their respective full-text manuscripts, especially in areas such as flow of participants and primary outcome data. These are not related to further recruitment of patients or word count limitations and are never explained.

**HOW THIS STUDY MIGHT AFFECT RESEARCH, PRACTICE OR POLICY**

- Caution is advised when abstract-only reports are used as evidence sources. Enhanced minimum reporting standards for abstract-only reports need to be employed.

## Introduction

 Randomised controlled trials (RCTs) are the gold standard for the assessment of efficacy of medical interventions.^1^ Systematic reviews (SRs) will often only include RCTs during the systematic search and evidence synthesis process, in order to eliminate biases and quality concerns related to other research designs. Recent years have seen an exponential increase in the number of SRs published, which use RCTs as their main source of evidence. SRs and RCTs are in turn prioritised in decision-making and the development of guideline recommendations in all medical fields, including gastroenterology.^2 3^

RCT findings are often initially published in abstract-only form, as proceedings of conferences of their respective medical fields, before the publication of a peer-reviewed full-text manuscript report in a scientific journal. These can be associated with press releases and interest, as well as presentations at major international meetings, often with the same work being presented repeatedly across the globe at different congresses to achieve maximum saturation of key stakeholders. Full-text manuscript publications undergo lengthy peer-review and editorial rounds, meaning that a number of years can pass between the publication of RCT findings in full-text manuscript form. It is also possible that a full-text manuscript may never come to fruition, in cases of publication bias, when final RCT findings are not what was initially expected. However, abstract-only reports are routinely used in evidence synthesis, during the period full-text manuscripts are not available, or when they do not exist. This is also key for guideline development and prescribing decision-making and given the risk of publication bias involved with excluding such works, the null position is very often to consider them.^4^ They are also used often by clinicians who want to stay updated with new findings in their field and prescribing decisions, and the press for the communication of these findings to the public. It has been demonstrated in other medical fields that abstract-only reports have both methodological and outcome data reporting flaws compared with full-text manuscripts. Abstract-only reports have been found to report data that are inconsistent with or absent from the full-text manuscript’s body, even in large-circulation general medical journals.^5^,^7^ Abstract-only reports can be at risk of substandard peer review,^8 9^ which could mean abstract outcome data used in evidence synthesis could be inaccurate. Additionally, methodological issues, such as whether allocation concealment has been reported or not, have been found to be associated with exaggerated effect estimates.^10^

Within the field of inflammatory bowel disease (IBD), methodological flaws have been identified even within full-text RCT manuscripts, with issues related to risk of bias^11^ and sample size estimation.^12 13^

Consistency of reporting between abstract-only and the respective full-text RCT manuscripts has not been investigated within the field of gastroenterology. The aim of this SR was to compare the concordance of prepublication abstract-only reports and their respective full-text manuscripts in outcome, attrition and magnitude of effect reporting for IBD RCTs, and to assess whether study authors provided justifications for potential mismatches.

## Methods

This review is reported in accordance with the Preferred Reporting Items for Systematic Reviews and Meta-Analyses.^14^ A protocol for the review was uploaded to an institutional online repository before commencement of data collection.^15^

### Inclusion and exclusion criteria

#### Full-text RCT manuscript selection

Inclusion criteria for full-text RCT manuscripts were as follows: (1) Full-text RCT manuscripts published between January 2010 and June 2023. The date range was chosen based on the year the Consolidated Standards of reporting trials (CONSORT) statement for the reporting of RCTs was reported until 6 months prior to data collection for this SR;^16^ (2) Full-text manuscripts of RCTs including patients with IBD of all age groups and any disease state or type; (3) Full-text manuscripts on RCT interventions for the induction, maintenance or management of symptoms, involving any pharmacological or non-pharmacological intervention compared with any other intervention, placebo, no treatment or usual care and (4) Full-text manuscripts which aimed to report the RCT’s protocol planned primary outcome data. There were no limitations on outcome measure, language or region.

Exclusion criteria were as follows: (1) manuscripts of non-randomised or quasi-randomised trials; (2) non-medical interventions such as service evaluation, delivery, safety, education and drug or symptom monitoring trials; (3) manuscripts without outcome results (eg, protocols); (4) manuscripts which only reported outcome data for non-randomised long-term RCT extensions and (5) manuscripts which only reported post hoc outcome data were excluded.

#### Abstract-only RCT report selection

Inclusion criteria for abstract-only RCT reports were as follows: (1) Abstract-only RCT reports published prior to the full-text trial RCT manuscript date; (2) Abstract-only reports of RCTs including patients with IBD of all age groups and any disease state or type; (3) Abstract-only reports on RCT interventions for the induction, maintenance or management of symptoms, involving any pharmacological or non-pharmacological intervention compared with any other intervention, placebo, no treatment or usual care and (4) Abstract-only reports which aimed to report the RCT’s protocol planned primary outcome data. Interim-results abstract-only reports were only included in the absence of finalised dataset reports. If more than one abstracts meeting the above criteria were identified, we included the abstract published closest to the date of publication of the full-text manuscript. If more than one abstract was published with similar text in different output forms, the first published abstract was included.

Exclusion criteria for abstract-only RCT reports matched those for full-text manuscript RCT reports.

### Screening and pairing of full-text manuscript to abstract-only reports

We conducted a systematic search via MEDLINE, EMBASE and CENTRAL for all potential full-text manuscripts and abstract-only reports (online supplemental appendix) in March 2022. An update search was conducted on July 2023. Search results were uploaded to a Microsoft Excel database for screening.

Screening and study selection took place in three stages. In the first stage (title screening), we discarded all duplicate and non-RCT reports. In the second stage (abstract screening), we identified reports which met our inclusion criteria, and separated them into two databases, one for full-text manuscripts and one for abstract-only reports. In the third stage (full-text screening), we identified all potential full-text manuscripts and abstract-only reports that met our criteria, and we formed pairs of full-text manuscripts and abstract-only reports for each RCT.

Eligibility of full-text RCT manuscripts was assessed by pairs of reviewers independently (AHAA-T, NS, AMAmE and SP) at all stages. Disagreement was resolved by discussion and consensus. A third author (MG or VS) resolved cases in which consensus was not reached.

Eligibility of abstract-only RCT reports was assessed in the same way, at all stages. Pairs of reviewers (AHAA-T, NS, SP and AMAmE) independently assessed all available abstract-only reports for eligibility and disagreement was resolved by discussion and consensus, or by a third author (MG or VS), when consensus could not be reached.

When an abstract-only report met the selection criteria, the assessors matched them with the full-text manuscript of the same RCT. Full-text RCT manuscripts and abstract-only RCT reports that were found to have no eligible matches at this stage were discarded.

When a single report was reporting on results of more than one RCTs (eg, randomised induction followed by a re-randomised maintenance of remission phase), we only considered relevant the parts of the report that were also reported in its matching pair. When both matching full-text manuscripts and abstract-only reports reported data from more than one RCTs, we included them twice (eg, as one induction pair and as one maintenance pair).

The systematic search and screening eliminated the risk of selection bias. Other forms of bias such as performance and detection bias were not relevant for the outcomes of this review, as they were not related to the effects of the performed interventions.

### Data extraction

Pairs of reviewers (AMAmE, ABR, SP and AHAA-T) independently performed data extraction using predesigned data extraction forms. A third author (MG or VS) resolved any disagreements.

The extracted reporting item data for full-text manuscript were the following: (1) Whether primary outcomes were stated; (2) Whether results for the stated primary outcomes were reported in any numerical form. If an outcome was reported but not stated whether it was primary or secondary, we considered all stated outcomes as primary; (3) Whether a trial registration was reported; (4) Whether a number of randomised patients per intervention group were reported; (5) Whether a number of participants reaching end of study per intervention group were reported; (6) Were the primary outcome data reported in a form where they could be used in a meta-analysis per intervention group and what were they; (7) What were the author conclusions (no difference, difference favouring intervention group or difference favouring control group) and (8) Explicit or implied justifications for the absence or non-concordance of any of the above items.

The extracted reporting item data for the matching abstract-only reports were the following: (1) Whether primary outcomes were stated and matched full-text; (2) Whether primary outcome results were reported in any form and matched full-text; (3) Whether a trial registration was reported and matched full text; (4) Whether a number of randomised patients per intervention group were reported and matched full-text; (5) Whether a number of participants reaching end of study per intervention group were reported and matched full-text; (6) Were the primary outcome data reported in a form where they could be used in a meta-analysis per intervention group and did they match the full-text; (7) What were the author conclusions (no difference, difference favouring intervention group or difference favouring control group) and if they matched full-text and (8) Explicit or implied justifications for the absence or non-concordance of any of the above items.

The reviewers chose a judgement of ‘yes’ when the reported item matched in both full-text manuscript and abstract-only publication, ‘no’ when the reported item did not match or that was not possible to determine due to unclear reporting in one of the two publications, or ‘missing’ when the item was not reported in both publications or it was not possible to determine due to unclear reporting in both publications, for each pair of full-text manuscript and abstract-only report, for each reporting item. Unclear reporting was ruled when there was not enough information to extract an item without contacting the authors. ‘No’ was only chosen when the reporting items clearly did not match, and not in cases of minimal mismatches, or when it was clear from context that the items matched.

AMAmE, ABR, SP and AHAA-T were not aware of the full-text manuscripts’ and abstract-only reports’ impact factor or abstract-only report’s source during data extraction.

After extraction of the reporting item data, publication characteristics for publication year, pharmacological or non-pharmacological intervention, impact factor of full-text manuscript and abstract-only report source were also extracted.

### Data analysis

We calculated descriptive statistics as absolute numbers and percentages for dichotomous outcomes, and mean (SD) for continuous. We conducted McNemar’s test for two related samples, with a binomial distribution used for missing data—when one of the two datapoints in a matching pair, for a given outcome, was missing—to test statistical differences between the abstract-only reports and full-text manuscripts. For pairs where both datapoints for a matching pair, for a given outcome, were missing, they were removed from the analysis of that outcome. We used SPSS V.28.0.

Subgroup analyses were performed for the following: period of publication (on or before 2016 vs 2017 or later—this was chosen as the midpoint between the beginning of our systematic search and when data analysis occurred); pharmacological versus non-pharmacological interventions; Impact factor of full-report journal (more or less than 20) and source of abstract-only report (different international conferences and meetings).

## Results

### Characteristics of included full-text manuscript and abstract-only report pairs

**Figure 1:**
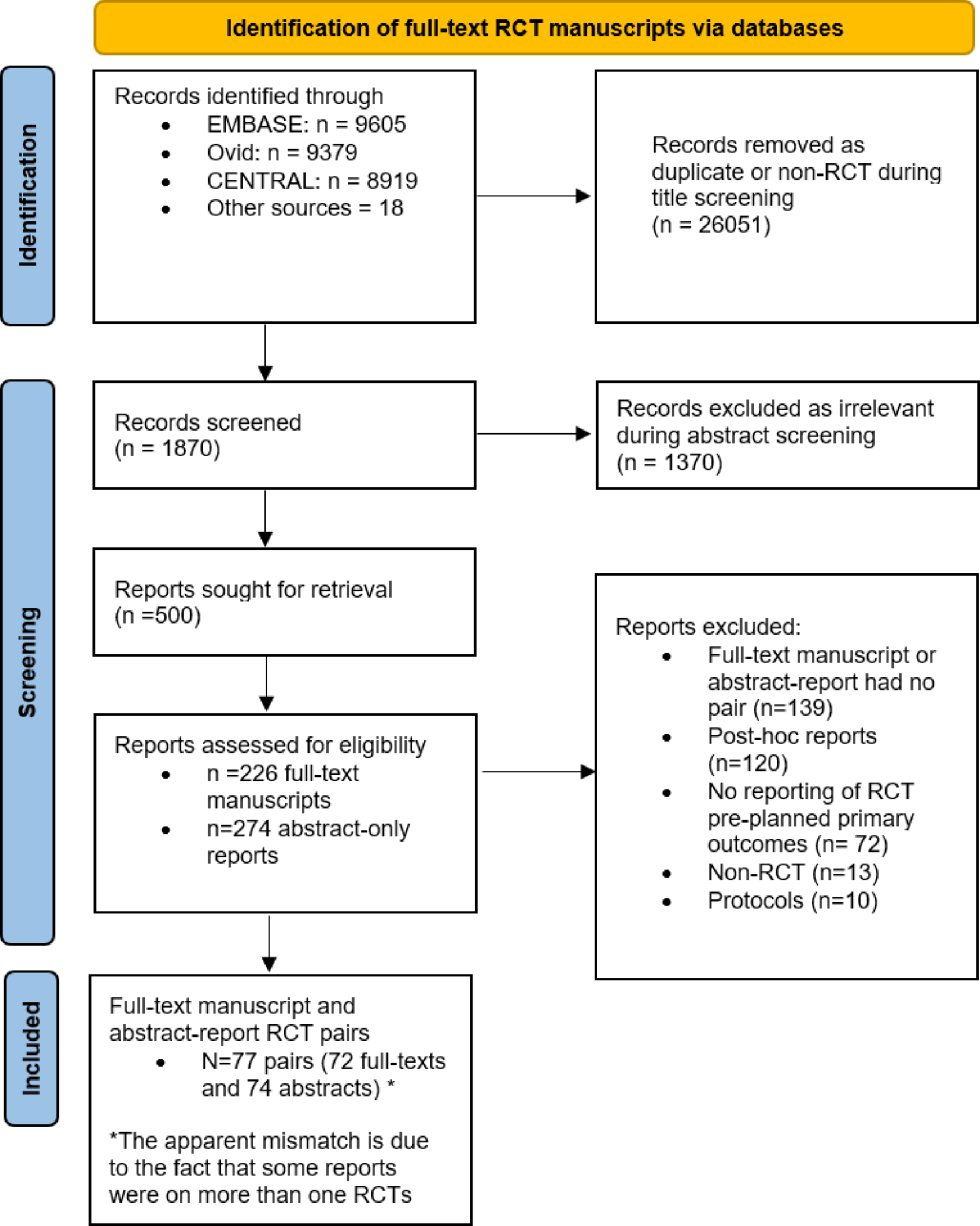
PRISMA flow diagram. RCTs, randomised controlled trials; PRISMA, Preferred reporting items for systematic reviews and meta-analysis.

The search and screening results are presented in figure 1. We were able to form 77 pairs of matching full-text manuscripts and abstract-only reports meeting our inclusion criteria (online supplemental file for references). Six full-text manuscripts and three abstract-only reports were included twice, as they reported results of separate RCTs (randomised induction of remission phases followed by re-randomised maintenance phases) (figure 1). The full list of references for all pairs is presented in online supplemental material. Descriptive characteristics are presented in table 1.

**Table 1:** Characteristics of included manuscripts and records

	Pharmacological/non-pharmacological	Year of publication of full-text manuscript	Year gap between full text and abstract	5-year impact factor of full-text journal	Impact factor more or less than 20	Abstract source (JCC, GastroJ, other)
Pairs, n (%) (total=77)	63 (82%) pharma 14 (17.9%) non-pharma	Reported for all	Reported for all	Retrieved for all	41 (53.8%) >20 36 (46.2%) <20	30 (38.5%) JCC 32 (42.3) GastroJ 15 (19.2) Other
Mean/median/mode	n/a	2017 (median) 2019 (mode)	1.19 (mean)	42.85 (mean)	n/a	n/a
Range/SD	n/a	2010–2022 (range)	1.1 (SD) 0–6 (range)	48.9 (SD) 0–130 (range)	n/a	n/a

GastroJ, *Gastroenterology Journal* (publishes the Digestive Diseases Week conferences’ abstracts); JCC, *Journal of Crohn’s and Colitis* (publishes the European Crohn's and Colitis Organisation’s conferences’ abstracts); n/a, not available.

#### Comparison of reporting items

**Figure 2:**
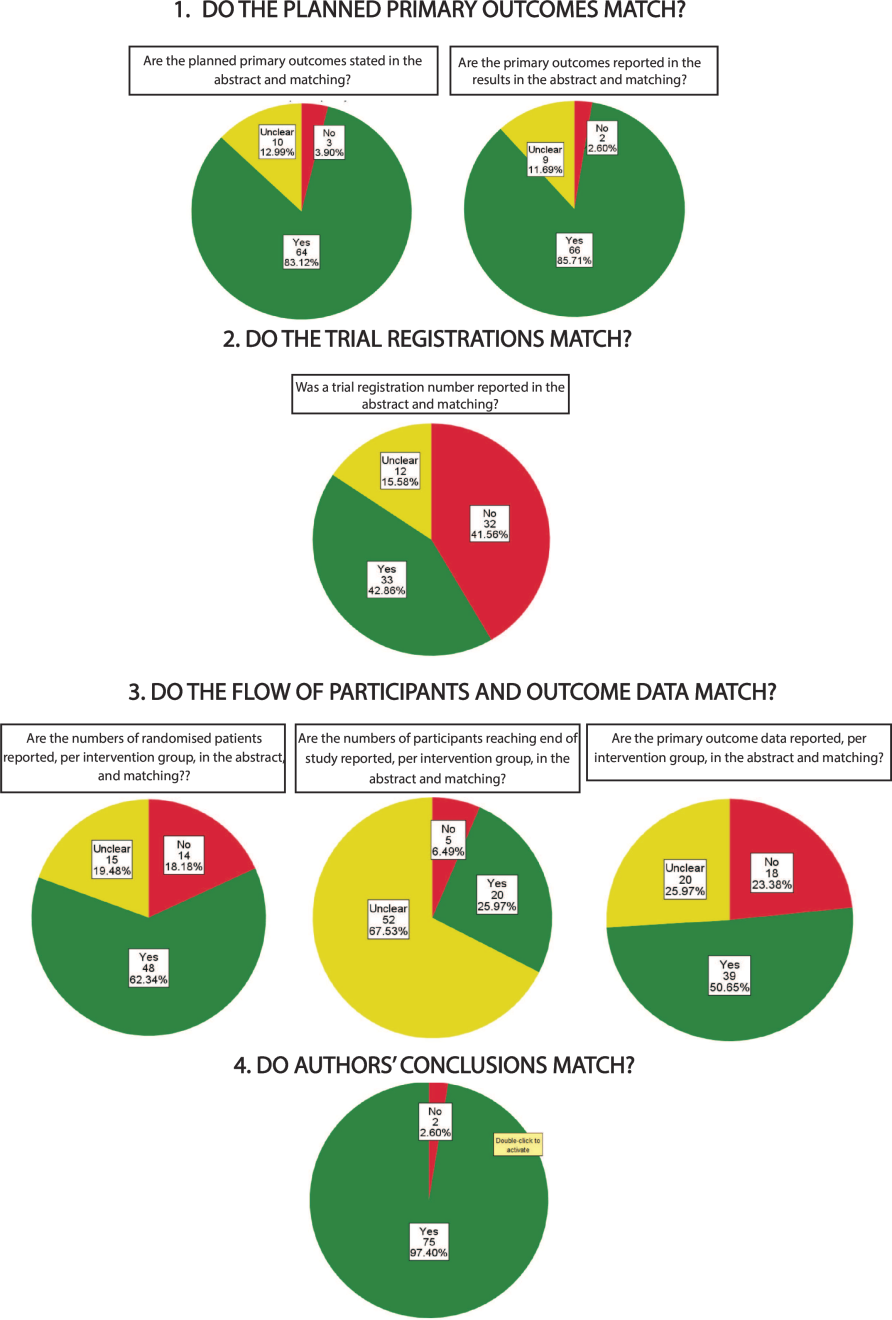
Reporting items compared between full-text manuscripts and abstract-only report pairs.

Figure 2 is a visual presentation of the results.

### Are the planned primary outcomes stated and reported?

Primary outcomes were stated in 76/77 full-text manuscripts (1 did not report) and matched in 64 abstract-only reports (3 were not matching, 10 did not report). Results for the stated primary outcomes were reported in any numerical form in 76/77 (1 did not report) full-text manuscripts and matched in 66 abstract-only reports (2 did not match, 9 did not report). Both reporting items were significantly mismatched (p<0.001, p=0.002).

### Are trial registrations reported?

Trial registrations were reported in 65/77 full-text manuscripts (13 did not report) and matched in 33 abstract-only reports (44 did not report). There was only one pair where a trial registration was reported in the abstract-only report and not in the full-text manuscript. This reporting item was significantly mismatched (p<0.001).

### Do the flow of participants and outcome data match?

The number of randomised patients per intervention group was reported in 76/77 full-text manuscripts (1 did not report) and matched in 48 abstract-only reports (14 did not match, 15 did not report).

The number of randomised participants reaching the end of the study was reported in 71/77 full-text manuscripts (9 did not report) and matched in 20 abstract-only reports (5 did not match, 52 did not report).

Outcome data per intervention group was reported in 73/77 full-text manuscripts (5 did not report) and matched in 39 abstract-only reports (18 did not match, 20 did not report).

The reporting of these items was significantly mismatched (p<0.001).

### Do authors’ conclusions match?

Author’s conclusions were reported in 77/77 full-text manuscripts and matched in 75 abstract-only reports (2 did not match, p=0.157).

### Are mismatch justifications provided?

We did not come across any instances of explicit or implied justifications for the absence or non-concordance for any of the items we compared between full-text manuscripts and abstract-only reports.

#### Subgroup analyses

For the 48 pairs with full-text manuscript published in 2017 and later, there was no difference from the main analysis. For the 29 pairs with full-text manuscript published in 2016 or earlier, there was no statistical difference for primary outcome stating and reporting (p=0.5, p=0.125). For the other items, there was no difference from the main analysis.

For the 63 pharmacological RCTs, there were no differences from the main results. For the 14 non-pharmacological RCTs there was no statistical difference for primary outcome stating and reporting, trial registration reporting, and reporting of randomised numbers (p=0.125, p=0.125, p=0.125, p=0.063). For the other items, there was no difference from the main analysis.

For the 42 pairs with impact factor of the full-text publication more than 20, there was no statistical difference for primary outcome stating (p=0.063). For the 36 pairs with impact factor of the full-text publication less than 20, there was no statistical difference for primary outcome reporting (p=0.125). For everything else, there was no difference from the main analysis.

For the 30 pairs with abstract-only reports published for the proceedings of the European Crohn's and Colitis Organisation (ECCO) conference, there was no statistical difference for primary outcome stating and reporting (p=0.063, p=0.125). For the 32 pairs with abstract-only reports published for the proceedings of the Digestive disease week (DDW) conference, there was no statistical difference for primary outcome stating and reporting (p=0.25, p=0.25). For the 15 pairs with abstract-only reports published elsewhere, there was no statistical difference for primary outcome stating and reporting (p=0.125, p=0.25). For everything else, there was no difference from the main analysis.

## Discussion

### Principal findings

We found significant mismatches and omissions between full-text manuscripts and prepublication abstract-only reports of IBD RCTs, for all reporting items apart from authors’ conclusions.

Our methodology has ensured these are not related to the use of different phases of a trial where data would not be expected to match, different follow-up time frames or even different subgroups and outcomes. Rather, the differences have remained unexplained by the authors, and in key areas which impact interpretation and associated quality assessment of the work.

The most stark finding is that in almost a quarter of paired texts, the main outcome data reported did not match for unexplained reasons (and for a further quarter it was unclear so a judgement could not even be made). Rather than obscure or experimental treatments, these paired publications included many treatments that are of significance to the field, often with much impact on practical prescribing level. Given the common use of these abstract texts for a significant period time by the international IBD community, this is a highly relevant finding that must be considered by all clinicians when reading such abstract forms of an RCT. While many of the differences could be explainable, the fact that this is not considered within the final publication and the abstract form is never amended or removed in the way that an RCT with incorrect data may be, forms a considerable risk to the use of such forms of evidence.

From a quality appraisal perspective, sparse or inconsistent reporting for randomised participant numbers and participant numbers reaching the end of an RCT, per intervention group, can increase the risk of reported attrition bias. For primary outcome data, it can increase the risk of selective reporting and misreporting of magnitude of effect. Insufficient statements for which outcomes an RCT planned to explore and which are actually reported, as well as for trial registration numbers in abstract-only reports, can also increase the risk of selective reporting, as it makes it difficult to ascertain whether the abstract reporting followed the plan of the RCT protocol. A common consideration is, of course, word count and readers may consider these requirements not feasible due to this reason. We believe that the minor clarification needed in several key areas is not explainable due to such issues. Rather, a lack of focus on the potential impact of such sparse reporting and how this echoes forward within the evidence base is a likely hypothesis, from both the perspective of the writer and the peer reviewer.

Combined, the issues of inconsistent key data and sparse quality reporting leave a significant source of evidence for clinicians, patients and wider stakeholders at significant risk when used in core activities to guide immediate and medium-term shifts in the ever-evolving portfolio of therapies for IBD.

Abstract-only reports are not always indexed in a fashion that highlights how they link to the many other forms of published outputs from the same single trial. This lack of a unified system that links together all published material for RCTs risks dual consideration of patient populations.

### Strengths and weaknesses of the present review

This is the first study of its kind to review the concordance between full-text manuscripts and abstract-only reports in gastroenterology RCTs. It highlights key reporting areas in need of improvement, which require minimal word space, which can improve risk of bias assessments, and increase quality of RCT reporting. In turn, this will increase the precision and consistency of evidence synthesis in subsequent SRs, guidelines and clinical decision-making. Due to the already mentioned issues with a lack of a unified method of identifying trials published in different mediums, there are possible matches of full-text manuscripts and abstract-only reports we might have missed. Additionally, there are more reporting items that can be compared in addition to the ones chosen that could further illuminate the issue.

### Strengths and weaknesses in relation to other studies

As far as we are aware, the topic of this review has not been reviewed in other medical areas by pairing full-text manuscripts and abstract-only reports. However, there have been other studies and reviews which have assessed the use of abstracts and abstract-only reports and their adherence to reporting standards and peer review, and have found them suboptimal^5^,^917^ A recent review on trustworthiness assessment for SRs found that 25% or RCTs included in 18 Cochrane SRs should not have been included in the reviews, due to issues around research governance, study feasibility and the plausibility of the results and reported baseline characteristics.^21^ The authors propose a formal trustworthiness assessment as part of the screening process and an inclusion criterion.

Our review focused on the role prepublication abstract-only reports play in SRs and guidelines, continuing previous work highlighting methodological flaws that can compromise evidence synthesis findings.^11^,^13^ Our findings add to the existing literature, suggesting there are serious issues with the trustworthiness of abstract reporting. Our subgroup analyses comparing newer to older RCTs suggest that abstract-only reporting of RCTs has may be only minimally improved with time. Further subgroup analyses suggest that whether an RCT is pharmacological or non-pharmacological, the impact factor of the full-text journal and the source of the abstract-only report, make no major difference.

Other studies could adopt our review approach in other fields gastroenterology or other medical fields, to establish the interpretability of our results more generally.

### Explanations and implications for clinicians and policy-makers

The implications for clinicians and policy-makers, who rely on SRs and guidelines for decision-making, is that the certainty of the available evidence is compromised, and timely updates of the evidence basis are difficult. The use of abstract-only reports is vital for evidence synthesis and updating, as they can be the only data source from RCTs that never get fully published due to publication bias, and they are the only available data source until a full-text manuscript gets published and becomes accessible. We found that it can take up to 6 years between the publication of an abstract-only report and its full-text manuscript, while sometimes a full-text manuscript never gets published. Even when an accessible full-text manuscript is available, abstract-only reports are still accessible and might be used instead. Our findings suggest, however, that abstract-only reporting is highly capricious and calls into question whether they should be trusted and used.

### Recommendations for the future

A number of simple practice changes could be implemented by different members of the research and clinical community. We believe the mandatory inclusion of trial registration numbers in all forms of trial presentation would allow easy cross-referencing and unification with little to no barriers to implementation. We also believe that the peer review systems for abstract-only publishing forums, predominately scientific meetings, could use their guidance documents or even checklists when RCTs are being submitted to ensure these bare minimum quality indices are reported. In the meantime, we would encourage all involved in evidence synthesis works and publishing reviews to consider comparisons that are mindful of the risk from abstract-only publication to ensure that risk is mitigated at the clinical practice level. Finally, editors and peer reviewers could ensure that previous abstracts are referenced in full-text publications and differences explained. This is key as it not only helps the clinical reader but also aids transparency is a key source of trial bias (selective reporting) further enhancing the quality of the trial publication. Research could produce a simple tool to aid this reporting for use across the field. At this point in time, guideline developers and policy-makers should not make recommendations based on abstract-only reporting. However, this can still occur, which makes our recommendations even more pertinent.

### Differences between protocol and review

We had originally planned to do meta-analyses for the continuous data we collected, however, we did not perform this due to the heterogeneity of the outcomes in the RCTs. We had also planned to do additional subgroup analyses for age (children vs adults), and primary outcome (induction vs maintenance vs other), however, these were not possible due to lack of adequate data. The subgroup analysis for pharmacological versus non-pharmacological interventions was planned retrospectively.

### Conclusions

Abstract-only reports were demonstrated to have consistent issues with both limited reporting of key information to judge quality and significant difference in data when compared with their later full-text publications. These are not related to further recruitment of patients or word count limitations and are never explained. As abstracts are often used by clinicians and in guidelines, reviews and stakeholder decision-making on prescribing, caution when using such sources of evidence is strongly suggested. Further work is needed to enhance minimum reporting standards in abstract-only works and ensure inconsistency with final published papers is highlighted.

## Supplementary material

10.1136/bmjgast-2023-001334 online supplemental file 1

## Data Availability

Data are available on reasonable request.
