# Supplementary material for: Prepublication abstract-only reports compared with full-text manuscripts for randomised controlled trials in inflammatory bowel disease: a systematic review
Source: BMJ Open Gastroenterol. 2024 Mar 7;11(1):e001334. doi: 10.1136/bmjgast-2023-001334 (PMC10921483; doi:10.1136/bmjgast-2023-001334)
Supplement: online supplemental file 1 [file bmjgast-2023-001334supp001.pdf]

Supplementary material. Full references of paired full-text manuscripts and abstract-only reports

**Study ID**	**Full-text reference**	**Pre-publication abstract-only reference**
Ankersen 2019	Ankersen, D.V., et al., *Individualized home-monitoring of disease activity in adult patients with inflammatory bowel disease can be recommended in clinical practice: A randomized-clinical trial.* World Journal of Gastroenterology, 2019. **25**(40): p. 6158.	Ankersen, D.V., et al., *DOP031 Individualised home-monitoring of disease activity in adult patients with inflammatory bowel disease can be recommended implemented in clinical practice.* Journal of Crohn's and Colitis, 2018. **12**(supplement_1): p. S052-S053
Armuzzi 2013	Armuzzi, A., et al., *Prevention of postoperative recurrence with azathioprine or infliximab in patients with Crohn's disease: an open-label pilot study.* Journal of Crohn's and Colitis, 2013. **7**(12): p. e623-e629	Armuzzi, A., et al., *Tu1231 Prevention of Postoperative Recurrence With Azathioprine or Anti-TNF Alpha in Patients With Crohn's Disease: an Open-Label Pilot Study.* Gastroenterology, 2012. **142**(5): p. S-780
Assa 2019 (PAILOT)	Assa, A., et al., *Proactive monitoring of adalimumab trough concentration associated with increased clinical remission in children with Crohn's disease compared with reactive monitoring.* Gastroenterology, 2019. **157**(4): p. 985-996. e2	Assa, A., et al., *OP18 Proactive adalimumab trough measurements increase corticosteroid-free clinical remission in paediatric patients with Crohn's disease: the paediatric Crohn's disease adalimumab-level-based optimisation treatment (PAILOT) trial.* Journal of Crohn's and Colitis, 2019. **13**(Supplement_1): p. S012-S013
Berding 2017	Berding, A., et al., *Beneficial effects of education on emotional distress, self-management, and coping in patients with inflammatory bowel disease: a prospective randomized controlled study.* Inflammatory intestinal diseases, 2016. **1**(4): p. 182-190	*P547. Effectiveness of patient education in inflammatory bowel disease.* Journal of Crohn's and Colitis, 2016. **10**(suppl_1): p. S378-S378
Buhl 2022	Buhl, Sine, et al. "Discontinuation of infliximab therapy in patients with Crohn's disease." *NEJM Evidence* 1.8 (2022): EVIDoa2200061.<br>[26]	*[27]* Buhl, Sine, et al. "742 DISCONTINUATION OF INFLIXIMAB THERAPY IN PATIENTS WITH CROHN'S DISEASE IN SUSTAINED, COMPLETE CLINICAL-BIOCHEMICAL-ENDOSCOPIC REMISSION: A DOUBLE-BLINDED, PLACEBO-CONTROLLED, RANDOMIZED CLINICAL TRIAL."

		Gastroenterology 160.6 (2021): S-151.
Carlsen 2017	Carlsen, K., et al., *Self-managed eHealth disease monitoring in children and adolescents with inflammatory bowel disease: a randomized controlled trial.* Inflammatory bowel diseases, 2017. **23**(3): p. 357-365	*DOP044. Self-administered telemedicine reduces number of outpatient visits and days of absence from school in paediatric and adolescent patients with inflammatory bowel disease.* Journal of Crohn's and Colitis, 2016. **10**(suppl_1): p. S53-S53
Chen 2020 (FERGIcor)	Chen, B., et al., *Efficacy and safety of adalimumab in Chinese patients with moderately to severely active Crohn's disease: results from a randomized trial.* Therapeutic advances in gastroenterology, 2020. **13**: p. 1756284820938960	Chen B, G.X., Zhong J, Ren J, Zhu X, Liu Z, Wu K, Kalabic J, Huang B, Doan T, Robinson AM, Chen M, *P0335 EFFICACY AND SAFETY OF ADALIMUMAB IN CHINESE PATIENTS WITH MODERATELY TO SEVERELY ACTIVE CROHN'S DISEASE.* United European Journal of Gastroenterology, 2018. **6**: p. A240
Colombel 2010 (SONIC)	Colombel, J.F., et al., *Infliximab, azathioprine, or combination therapy for Crohn's disease.* New England journal of medicine, 2010. **362**(15): p. 1383-1395	Colombel, J., P. Rutgeerts, and W. Reinisch, *One year data from the sonic study: A randomized, double-blind trial comparing infliximab and infliximab plus azathioprine to azathioprine in patients with Crohn's disease naive to immunomodulators and biologic therapy.* Gut, 2009. **58**(Suppl II): p. A69
Cosnes 2013	Cosnes, J., et al., *Early administration of azathioprine vs conventional management of Crohn's disease: a randomized controlled trial.* Gastroenterology, 2013. **145**(4): p. 758-765. e2.	Cosnes, J., A. Bourrier, and Y. Bouhnik, *Accelerated step-care therapy with early azathioprine vs. conventional step-care therapy in Crohn's disease. A randomized study.* Gastroenterology, 2012. **142**: p. S161
Cross 2012 (UC-HAT)	Cross, R.K., et al., *Randomized, controlled trial of home telemanagement in patients with ulcerative colitis (UC HAT).* Inflammatory bowel diseases, 2012. **18**(6): p. 1018-1025	Cross, R., et al., *T1240 A Randomized, Controlled Trial of Home Telemanagement in Patients With Ulcerative Colitis (UC HAT).* Gastroenterology, 2010. **138**(5): p. S-519
D'Haens 2022 (ADVANCE)	D'Haens, Geert, et al. "Risankizumab as induction therapy for Crohn's disease: results from the phase 3 ADVANCE and MOTIVATE induction trials." *The Lancet* 399.10340 (2022): 2015-2030.	D'Haens, Geert R., et al. "775a Risankizumab induction therapy in patients with moderate-to-severe Crohn's disease with intolerance or inadequate

	[38]	response to conventional and/or biologic therapy: results from the phase 3 ADVANCE study." *Gastroenterology* 161.2 (2021): e28.[39]
D'Haens 2022 (MOTIVATE)	D'Haens, Geert, et al. "Risankizumab as induction therapy for Crohn's disease: results from the phase 3 ADVANCE and MOTIVATE induction trials." *The Lancet* 399.10340 (2022): 2015-2030	Panaccione, Remo, et al. "S754 Risankizumab as induction therapy in patients with moderately to severely active Crohn's disease who failed 1 vs> 1 prior biologic treatment: results from the MOTIVATE study." *Official journal of the American College of Gastroenterology| ACG* 116 (2021): S348.[40]
D'Haens 2023 (LUCENT)	[41] D'Haens, Geert, et al. "Mirikizumab as induction and maintenance therapy for ulcerative colitis." *New England Journal of Medicine* 388.26 (2023): 2444-2455.	*Andrews, J. M., et al. "Efficacy and safety of mirikizumab as induction therapy in patients with moderately to severely active ulcerative colitis: Results from the Phase 3 LUCENT-1 study." JOURNAL OF GASTROENTEROLOGY AND HEPATOLOGY. Vol. 37. 111 RIVER ST, HOBOKEN 07030-5774, NJ USA: WILEY, 2022.[42]*
Danese 2017 (ANDANTE - induction)	Danese, S., et al., *Randomised trial and open-label extension study of an anti-interleukin-6 antibody in Crohn's disease (ANDANTE I and II).* Gut, 2019. **68**(1): p. 40-48	*OP015. Results of ANDANTE, a randomised clinical study with an anti-IL6 antibody (PF-04236921) in subjects with Crohn's disease who are anti-tumour necrosis factor inadequate responders.* Journal of Crohn's and Colitis, 2016. **10**(suppl_1): p. S12-S13.
Del Hoyo 2018 (TECCU)	Del Hoyo, J., et al., *A web-based telemanagement system for improving disease activity and quality of life in patients with complex inflammatory bowel disease: pilot randomized controlled trial.* Journal of medical Internet research, 2018. **20**(11): p. e11602	Aguas, M., et al., *P227 A web-based telemanagement system for patients with complex Inflammatory Bowel Disease (TECCU): Results of a randomised controlled clinical trial.* Journal of Crohn's and Colitis, 2018. **12**(supplement_1): p. S214-S215
Evstatiev 2011	Evstatiev, R., et al., *FERGIcor, a randomized controlled trial on ferric carboxymaltose for iron deficiency anemia in inflammatory bowel disease.* Gastroenterology, 2011. **141**(3): p. 846-853. e2	Evstatiev, R., et al., *Intravenously administered ferric carboxymaltose and iron sucrose significantly improve quality of life in patients with IBD-associated iron deficiency*

		anaemia. J Crohns Colitis, 2011. **5**: p. S91.
Feagan 2013 (GEMINI 1-induction)	Feagan, B.G., et al., *Vedolizumab as induction and maintenance therapy for ulcerative colitis.* New England Journal of Medicine, 2013. **369**(8): p. 699-710	Feagan, B.G., et al., *943b Induction Therapy for Ulcerative Colitis: Results of GEMINI I, a Randomized, Placebo-Controlled, Double-Blind, Multicenter Phase 3 Trial.* Gastroenterology, 2012. **142**(5): p. S-160-S-161
Feagan 2013 (GEMINI 1 - maintenance)	Feagan, B.G., et al., *Vedolizumab as induction and maintenance therapy for ulcerative colitis.* New England Journal of Medicine, 2013. **369**(8): p. 699-710	Feagan, B., et al., *Vedolizumab Maintenance Therapy for Ulcerative Colitis: Results of GEMINI I, a Randomized, Placebo-Controlled, Double-Blind, Multicenter Phase 3 Trial: 1522.* Official journal of the American College of Gastroenterology| ACG, 2012. **107**: p. S609-S610
Feagan 2014	Feagan, B.G., et al., *Methotrexate in combination with infliximab is no more effective than infliximab alone in patients with Crohn's disease.* Gastroenterology, 2014. **146**(3): p. 681-688. e1	Feagan, B., et al., *A randomized trial of methotrexate in combination with infliximab for the treatment of Crohn's disease.* Gastroenterology, 2008. **135**(1): p. 294-295
Feagan 2017	Feagan, B.G., et al., *Induction therapy with the selective interleukin-23 inhibitor risankizumab in patients with moderate-to-severe Crohn's disease: a randomised, double-blind, placebo-controlled phase 2 study.* The Lancet, 2017. **389**(10080): p. 1699-1709	Feagan, B., et al., *Efficacy and safety of induction therapy with the selective IL-23 inhibitor risankizumab (BI 655066), in patients with moderate-to-severe Crohn's disease: results of a randomized, double-blind, placebo-controlled phase II study.* Digestive Disease Week, San Diego, USA, 2016.
Flourié 2013 (MOTUS)	*Randomised clinical trial: once-vs. twice-daily prolonged-release mesalazine for active ulcerative colitis.* Alimentary pharmacology & therapeutics, 2013. **37**(8): p. 767-775	Flourie, B., et al., *1097 Once-Daily Versus Twice-Daily Mesalazine for Active Ulcerative Colitis: Efficacy Results From MOTUS, a Multicentre, Controlled, Randomised, Investigator-Blinded Study.* Gastroenterology, 2012. **5**(142): p. S-197
Gasche 2015	Gasche, C., et al., *Ferric maltol is effective in correcting iron deficiency anemia in patients with inflammatory bowel disease: results from a phase-3 clinical trial program.* Inflammatory bowel diseases, 2015. **21**(3): p. 579-588	Gasche, C., et al., *DOP079 Correcting iron deficiency anaemia in IBD: A pivotal phase 3 study of a novel oral ferric iron.* Journal of Crohn's and

		Colitis, 2014. **8**(Supplement_1): p. S53-S53.
Hanauer 2021 (VOLTAIRE-CD)	Hanauer, S., et al., *Safety and efficacy of BI 695501 versus adalimumab reference product in patients with advanced Crohn's disease (VOLTAIRE-CD): a multicentre, randomised, double-blind, phase 3 trial.* The Lancet Gastroenterology & Hepatology, 2021. **6**(10): p. 816-825.	[61] Hanauer, S.B., et al., *Tu1862 BI 695501 DEMONSTRATES SIMILAR EFFICACY AND COMPARABLE SAFETY TO ADALIMUMAB REFERENCE PRODUCT IN PATIENTS WITH ACTIVE CROHN'S DISEASE: FINAL ANALYSIS OF THE PHASE III VOLTAIRE-CD STUDY.* Gastroenterology, 2020. **158**(6): p. S-1192-S-1193
Hawthorne 2012	Hawthorne, B.A., et al., *One-year investigator-blind randomized multicenter trial comparing Asacol 2.4 g once daily with 800 mg three times daily for maintenance of remission in ulcerative colitis.* Inflammatory Bowel Diseases, 2012. **18**(10): p. 1885-1893.	Hawthorne, A.B., et al., *Once daily mesalazine as maintenance therapy for ulcerative colitis (UC): a one-year single-blind randomised trial.* Gastroenterology, 2011. **5**(140): p. S-65
Heida 2018	Heida, A., et al., *Efficacy of home telemonitoring versus conventional follow-up: a randomized controlled trial among teenagers with inflammatory bowel disease.* Journal of Crohn's and Colitis, 2018. **12**(4): p. 432-441.	Heida A, D.A., Muller Kobold A, Kindermann A, Kokke F, de Meij T, Norbruis O, Wessels M, Hummel T, Escher H, van Wering H, Hendriks D, Mearin L, Groen H, Verkade H, van Rheenen P, *OP05. Telemonitoring versus usual care: a multicenter trial among teenagers with inflammatory bowel disease.* 4th International Symposium on Pediatric Inflammatory Bowel Disease, 2017. **65**: p. S3
Herfarth 2018 (MERIT-UC)	Herfarth, H., et al., *Methotrexate is not superior to placebo in maintaining steroid-free response or remission in ulcerative colitis.* Gastroenterology, 2018. **155**(4): p. 1098-1108. e9	Herfarth, H., et al., *P390 Methotrexate is not superior to placebo in maintaining remission in patients with ulcerative colitis: results from the MERIT-UC study.* Journal of Crohn's and Colitis, 2018. **12**(supplement_1): p. S300-S301
Howaldt 2022	Howaldt, Stefanie, et al. "Long-Term effectiveness of oral ferric maltol vs intravenous ferric carboxymaltose for the treatment of iron-deficiency anemia in patients with inflammatory bowel disease: a randomized controlled Noninferiority trial."	Howaldt, S., et al. "Oral ferric maltol versus intravenous ferric carboxymaltose for the treatment of iron-deficiency anaemia in patients with inflammatory bowel disease: a multicentre phase 3b, open-

	Inflammatory Bowel Diseases 28.3 (2022): 373-384.[68]	label randomised controlled trial." *United European Gastroenterol J* 7 (2019).[69]
Jørgensen 2017 (NOR-SWITCH)	Jørgensen, K.K., et al., *Switching from originator infliximab to biosimilar CT-P13 compared with maintained treatment with originator infliximab (NOR-SWITCH): a 52-week, randomised, double-blind, non-inferiority trial.* The Lancet, 2017. **389**(10086): p. 2304-2316	Goll GL, O.I., Jorgensen KK, Lorentzen M, Bolstad N, Haavardsholm EA, Lundin KE, Mork C, Jahnsen J, Kvien TK, *Biosimilar Infliximab (CT-P13) Is Not Inferior to Originator Infliximab: Results from a 52-Week Randomized Switch Trial in Norway [abstract].* Arthritis Rheumatol, 2016. **68**
Lawrance 2017	Lawrance, I.C., et al., *Efficacy of rectal tacrolimus for induction therapy in patients with resistant ulcerative proctitis.* Clinical Gastroenterology and Hepatology, 2017. **15**(8): p. 1248-1255	Lawrance, I.C., et al., *OP007 A multi-centre double blind randomised placebo-controlled study of the use of rectal tacrolimus in the treatment of resistant ulcerative proctitis.* Journal of Crohn's and Colitis, 2017. **11**(suppl_1): p. S4-S5
Levine 2019	Levine, A., et al., *Crohn's disease exclusion diet plus partial enteral nutrition induces sustained remission in a randomized controlled trial.* Gastroenterology, 2019. **157**(2): p. 440-450. e8	van Limbergen, J.E., et al.*; Crohn's Disease Exclusion Diet is Equally Effective But Better Tolerated Than Exclusive Enteral Nutrition for Induction of Remission in Mild-To-Moderate Pediatric Crohn's Disease: A Prospective Randomized Controlled Trial.* Gastroenterology, 2019. **156**(6): p. S-59
Lie 2020	Lie, M.R., et al., *No superiority of tacrolimus suppositories vs beclomethasone suppositories in a randomized trial of patients with refractory ulcerative proctitis.* Clinical Gastroenterology and Hepatology, 2020. **18**(8): p. 1777-1784. e2	Kreijne, J.E., et al., *DOP022 Tacrolimus suppositories as induction therapy for refractory ulcerative proctitis: a randomised controlled trial.* Journal of Crohn's and Colitis, 2018. **12**(supplement_1): p. S045-S045
Luglio 2020 (SuPREMe-CD)	Luglio, G., et al., *Surgical prevention of anastomotic recurrence by excluding mesentery in Crohn's disease: the SuPREMe-CD study-a randomized clinical trial.* Annals of surgery, 2020. **272**(2): p. 210-217	Luglio, G., et al., *OP18 Surgical prevention of anastomotic recurrence by excluding mesentery in Crohn's disease: The SuPREMe-CD study.* Journal of Crohn's and Colitis, 2020. **14**(Supplement_1): p. S015-S016
Mañosa 2013	Mañosa, M., et al., *Addition of metronidazole to azathioprine for the prevention of postoperative recurrence of*	Mañosa, M., et al., *P210 Azathioprine versus azathioprine plus metronidazole*

	Crohn's disease: a randomized, double-blind, placebo-controlled trial. Inflammatory Bowel Diseases, 2013. **19**(9): p. 1889-1895	*for the prevention of postoperative endoscopic recurrence of Crohn's disease: A randomized, placebo-controlled trial.* Journal of Crohn's and Colitis, 2012. **6**(Supplement_1): p. S93-S93
Matsumoto 2016	Matsumoto, Takayuki, et al. "Adalimumab monotherapy and a combination with azathioprine for Crohn's disease: a prospective, randomized trial." *Journal of Crohn's and Colitis* 10.11 (2016): 1259-1266. [82]	[83] Matsumoto, T., et al. "Comparison of adalimumab monotherapy and a combination with azathioprine for patients with Crohn's disease: a prospective, multicentre, open-labelled clinical trial (DIAMOND study)." *J Crohns Colitis* 10 (2016): S8.
McCombie 2020	McCombie, A., et al., *A noninferiority randomized clinical trial of the use of the smartphone-based health applications IBDsmart and IBDoc in the care of inflammatory bowel disease patients.* Inflammatory bowel diseases, 2020. **26**(7): p. 1098-1109	Walmsley, R., et al., *P630 A non-inferiority randomised clinical trial of the use of the smartphone-based health applications IBDsmart and IBDoc® in the care of inflammatory bowel disease patients.* Journal of Crohn's and Colitis, 2019. **13**(Supplement_1): p. S432-S433
Mowat 2016 (TOPPIC)	Mowat, C., et al., *Mercaptopurine versus placebo to prevent recurrence of Crohn's disease after surgical resection (TOPPIC): a multicentre, double-blind, randomised controlled trial.* The lancet Gastroenterology & hepatology, 2016. **1**(4): p. 273-282	Arnott, I., et al. *The TOPPIC Trial: a randomised, double-blind parallel-group trial of mercaptopurine versus placebo to prevent recurrence of Crohn's disease following surgical resection in 240 patients*. in *JOURNAL OF CROHNS & COLITIS*. 2016. OXFORD UNIV PRESS GREAT CLARENDON ST, OXFORD OX2 6DP, ENGLAND
Motoya 2019 (induction)	Motoya, S., et al., *Vedolizumab in Japanese patients with ulcerative colitis: a phase 3, randomized, double-blind, placebo-controlled study.* PLoS One, 2019. **14**(2): p. e0212989	Watanabe, M., et al., *Sa1751 - A Phase 3 Study of Vedolizumab for Induction and Maintenance Therapy in Japanese Patients with Moderately to Severely Active Ulcerative Colitis.* Gastroenterology, 2018. **154**(6): p. S-380-S-381
Motoya 2019 (maintenance)	Motoya, S., et al., *Vedolizumab in Japanese patients with ulcerative colitis: a phase 3, randomized, double-blind, placebo-*	Watanabe, M., et al., *Sa1751 - A Phase 3 Study of Vedolizumab for Induction and Maintenance Therapy in Japanese Patients*

	controlled study. PLoS One, 2019. **14**(2): p. e0212989	*with Moderately to Severely Active Ulcerative Colitis.* Gastroenterology, 2018. **154**(6): p. S-380-S-381
Naganuma 2017 (INDIGO)	Naganuma, M., et al., *Efficacy of indigo naturalis in a multicenter randomized controlled trial of patients with ulcerative colitis.* Gastroenterology, 2018. **154**(4): p. 935-947.	Naganuma M, S.S., Mitsuyama K, Kobayashi T, Yoshimura N, Matsuoka K, Hisamatsu T, Watanabe K, Abe T, Suzuki Y, Hibi T, Kanai T, *OP098 INDIGO NATURALIS IS EFFECTIVE FOR INDUCING CLINICAL REMISSION AND MUCOSAL HEALING IN PATIENTS WITH ULCERATIVE COLITIS (INDIGO STUDY).* United European Journal of Gastroenterology, 2017. **5**: p. A41-2
Nikolaus 2017	Nikolaus, S., et al., *Patient education in a 14-month randomised trial fails to improve adherence in ulcerative colitis: influence of demographic and clinical parameters on non-adherence.* Journal of Crohn's and Colitis, 2017. **11**(9): p. 1052-1062	Nikolaus, S., et al., *DOP044 Patient education in a 14 month randomized trial fails to improve adherence in ulcerative colitis: Influence of demographic and clinical parameters on non-adherence.* Journal of Crohn's and Colitis, 2014(8): p. S36
Ozgursoy-Uran 2019	Uran, B.N.O., S. Aykar, and Y. Yildirim, *The effect of web-based education on disease activity, symptom management and quality of life in patients with inflamatory bowel disease: randomized-controlled study.* Medical Science, 2019. **23**(98): p. 415-431	Ozgursoy Uran, B.N., et al., *N031 The effect of web-based education on disease activity, symptom management, and quality of life on patients with inflammatory bowel disease.* Journal of Crohn's and Colitis, 2018. **12**(supplement_1): p. S582-S582
Panes 2017	Panés, J., et al., *Tofacitinib for induction and maintenance therapy of Crohn's disease: results of two phase IIb randomised placebo-controlled trials.* Gut, 2017. **66**(6): p. 1049-1059.	Panés, J., et al., *855 Efficacy and Safety of Tofacitinib for Oral Induction Therapy in Patients With Moderate to Severe Crohn's Disease: Results of a Phase 2B Randomized Placebo-Controlled Trial.* Gastroenterology, 2016. **150**(4): p. S182-S183.
Panes 2022	Panés, J., Colombel, J. F., D'Haens, G. R., Schreiber, S., Panaccione, R., Peyrin-Biroulet, L., ... & Sandborn, W. J. (2022). Higher vs standard adalimumab induction and maintenance dosing regimens for treatment of ulcerative colitis: SERENE UC trial results. *Gastroenterology*, *162*(7), 1891-1910.	[99] Colombel, J. F., et al. "OP01 Higher vs. standard adalimumab maintenance regimens in patients with moderately to severely active ulcerative colitis: Results from the SERENE-UC maintenance study." *Journal of Crohn's and Colitis*

	[98]	14.Supplement_1 (2020): S001-S001.
Park 2019	Park, S.-K., et al., *Adherence to Asacol once daily versus divided regimen for maintenance therapy in ulcerative colitis: a prospective, multicenter, randomized study.* Intestinal research, 2019. **17**(3): p. 349-356	Park, S.-K., et al., *P517 The effects and adherence of Asacol® comparing 2.4 g once daily with 800 mg three times or 1200 mg twice daily for maintain therapy in the ulcerative colitis: Prospective multicentre randomised study.* Journal of Crohn's and Colitis, 2018. **12**(supplement_1): p. S367-S367
Petersen 2014	Petersen, A.M., et al., *Ciprofloxacin and probiotic Escherichia coli Nissle add-on treatment in active ulcerative colitis: a double-blind randomized placebo controlled clinical trial.* Journal of Crohn's and Colitis, 2014. **8**(11): p. 1498-1505	Petersen, A.M., et al., *Mo1227 Ciprofloxacin and Probiotic Escherichia coli Nissle As Add-on Treatment in Active Ulcerative Colitis; a Double-Blinded Randomized Placebo Controlled Clinical Trial.* Gastroenterology, 2014. **146**(5): p. S-591
Reich 2019 (Mychart-Epic)	Reich, J., et al., *The use of An EHR patient portal (Mychart-Epic) in patients with inflammatory bowel disease.* Crohn's & Colitis 360, 2019. **1**(3): p. otz039.	Reich, J., et al., *The Use of an EMR Patient Portal (MYCHART-EPIC) in Patients With Inflammatory Bowel Disease: Preliminary Results of a Randomized Clinical Trial: 609.* Official journal of the American College of Gastroenterology| ACG, 2018. **113**: p. S349
Roblin 2019	Roblin, X., et al., *Addition of azathioprine to the switch of anti-TNF in patients with IBD in clinical relapse with undetectable anti-TNF trough levels and antidrug antibodies: a prospective randomised trial.* Gut, 2020. **69**(7): p. 1206-1212	Roblin, X., et al., *345 - Interest in the Addition of Azathioprine (AZA) to the Switch of Anti-TNF in IBD Patients in Clinical Relapse with Undetectable Anti-TNF trough Levels and Anti-Drug Antibodies: A Prospective Randomized Trial.* Gastroenterology, 2018. **154**(6): p. S-84.
Rutgeerts 2012 (EXTEND)	Rutgeerts, P., et al., *Adalimumab induces and maintains mucosal healing in patients with Crohn's disease: data from the EXTEND trial.* Gastroenterology, 2012. **142**(5): p. 1102-1111. e2.	Colombel, J.-F., et al., *T1239 Adalimumab Treatment Results in Deep Remission for Patients With Moderate to Severe Ileocolonic Crohn's Disease: Results*

		From EXTEND. Gastroenterology, 2010. **138**(5): p. S-518.
Sandborn 2010 (WELCOME)	Sandborn, W.J., et al., *Certolizumab pegol in patients with moderate to severe Crohn's disease and secondary failure to infliximab.* Clinical Gastroenterology and Hepatology, 2010. **8**(8): p. 688-695. e2	Sandborn, W.J., et al., *143 Welcome: A Randomized, Double-Blind, Controlled Trial Comparing Certolizumab Pegol 400 Mg Every 2 Weeks with Every 4 Weeks for Maintenance of Response and Remission in Patients with Moderate to Severe Crohn's Disease with Secondary Failure to Infliximab.* Gastroenterology, 2009. **5**(136): p. A-27
Sandborn 2011	Sandborn, W.J., et al., *Certolizumab pegol for active Crohn's disease: a placebo-controlled, randomized trial.* Clinical gastroenterology and hepatology, 2011. **9**(8): p. 670-678. e3	Sandborn, W., et al., *Induction Therapy with Certolizumab Pegol in Patients with Moderate to Severe Crohn's Disease: A Placebo-Controlled Trial: 1156.* Official journal of the American College of Gastroenterology| ACG, 2010. **105**: p. S419
Sandborn 2012	Sandborn, W.J., et al., *Once-daily budesonide MMX® extended-release tablets induce remission in patients with mild to moderate ulcerative colitis: results from the CORE I study.* Gastroenterology, 2012. **143**(5): p. 1218-1226. e2	Sandborn, W.J., et al., *Budesonide Mxx® 9 mg for the Induction of Remission of Mild-to-Moderate Ulcerative Colitis (UC): Data From a Multicenter, Randomized, Double-Blind Placebo-Controlled Study in North America and India.* Gastroenterology, 2011. **5**(140): p. S-124.
Sandborn 2012 (CERTIFI-induction)	Sandborn, W.J., et al., *Ustekinumab induction and maintenance therapy in refractory Crohn's disease.* New England Journal of Medicine, 2012. **367**(16): p. 1519-1528	Sandborn, W.J., et al., *A multicenter, randomized, double-blind, placebo-controlled phase2b Study or Ustekinumab, a human monoclonal antibody to IL-12/23p40, in patients with moderately to severely active Crohn's disease: results through week 22 from the certifi trial.* Gastroenterology, 2011. **140**(5): p. S109-S109
Sandborn 2012 (CERTIFI-maintenance)	Sandborn, W.J., et al., *Ustekinumab induction and maintenance therapy in refractory Crohn's disease.* New England Journal of Medicine, 2012. **367**(16): p. 1519-1528	Sandborn, W.J., et al., *A multicenter, randomized, double-blind, placebo-controlled phase2b Study or Ustekinumab, a human monoclonal antibody to IL-12/23p40, in patients with*

		moderately to severely active Crohn's disease: results through week 22 from the certifi trial. Gastroenterology, 2011. **140**(5): p. S109-S109
Sandborn 2013 (GEMINI 2-induction)	Sandborn, W.J., et al., *Vedolizumab as induction and maintenance therapy for Crohn's disease.* New England Journal of Medicine, 2013. **369**(8): p. 711-721	Hanauer, S.B., et al., *Tu1138 Efficacy of Vedolizumab in Crohn's disease by prior treatment failure in Gemini II, a randomized, placebo-controlled, double-blind, multicenter study.* Gastroenterology, 2013. **5**(144): p. S-772.
Sandborn 2013 (GEMINI 2maintenance)	Sandborn, W.J., et al., *Vedolizumab as induction and maintenance therapy for Crohn's disease.* New England Journal of Medicine, 2013. **369**(8): p. 711-721	Rutgeerts, P.J., et al., *Sustained Therapeutic Benefit of Vedolizumab Throughout 1 Year in Crohn's Disease in Gemini II, a Randomized, Placebo-Controlled, Double-Blind, Multicenter Study.* Gastroenterology, 2013. **144**(5): p. S21-S21
Sandborn 2019	Sandborn, W.J., et al., *Efficacy and safety of mirikizumab in a randomized phase 2 study of patients with ulcerative colitis.* Gastroenterology, 2020. **158**(3): p. 537-549. e10.	Sandborn, W.J., et al., *882-Efficacy and safety of anti-interleukin-23 therapy with mirikizumab (Ly3074828) in patients with moderate-to-severe ulcerative colitis in a phase 2 study.* Gastroenterology, 2018. **154**(6): p. S-1360-S-1361
Sandborn 2020 (CELEST)	[123] Sandborn, W.J., et al., *Efficacy and safety of upadacitinib in a randomized trial of patients with Crohn's disease.* Gastroenterology, 2020. **158**(8): p. 2123-2138. e8	Panes, J., et al., *P273 Efficacy and safety of upadacitinib maintenance treatment for moderate to severe Crohn's disease: Results from the CELEST study.* Journal of Crohn's and Colitis, 2018. **12**(supplement_1): p. S238-S239
Sandborn 2022 (GALAXI-1)	Sandborn, W.J., et al., *Guselkumab for the treatment of Crohn's disease: induction results from the phase 2 GALAXI-1 study.* Gastroenterology, 2022. **162**(6): p. 1650-1664. e8.	Sandborn, W., et al., *The efficacy and safety OF guselkumab induction therapy IN patients with moderately to severely active CROHN'S disease: week 12 interim analyses from the phase 2 GALAXI 1 study.* United Eur

		Gastroenterol J, 2020. **8**(8S): p. 64.
Sandborn 2022 (BERGAMOT Cohort 1)	[127] Sandborn, William J., et al. "Etrolizumab as induction and maintenance therapy in patients with moderately to severely active Crohn's disease (BERGAMOT): a randomised, placebo-controlled, double-blind, phase 3 trial." *The Lancet Gastroenterology & Hepatology* 8.1 (2023): 43-55.	William, Sandborn, et al. "Etrolizumab as Induction Therapy in Moderate to Severe Crohn's Disease: Results From BERGAMOT Cohort 1: P-011." *Official journal of the American College of Gastroenterology| ACG* 113 (2018): S3. [128]
Sands 2014 (GEMINI 3)	Sands, B.E., et al., *Effects of vedolizumab induction therapy for patients with Crohn's disease in whom tumor necrosis factor antagonist treatment failed.* Gastroenterology, 2014. **147**(3): p. 618-627. e3	Sands, B., et al., *11 Vedolizumab induction therapy for patients with Crohn's disease and prior anti-tumour necrosis factor antagonist failure: a randomised, placebo-controlled, double-blind, multicentre trial.* Journal of Crohn's and Colitis, 2013. **7**(Supplement_1): p. S5-S6.
Sands 2019 (UNIFI)	Sands, B.E., et al., *Ustekinumab as induction and maintenance therapy for ulcerative colitis.* New England Journal of Medicine, 2019. **381**(13): p. 1201-1214.	Sands, B., et al., *Safety and efficacy of ustekinumab induction therapy in patients with moderate to severe ulcerative colitis: results from the Phase 3 UNIFI study.* United European Gastroenterol J, 2018. **6**(8 suppl).
Sands 2019 (VARSITY)	Sands, B.E., et al., *Vedolizumab versus adalimumab for moderate-to-severe ulcerative colitis.* New England Journal of Medicine, 2019. **381**(13): p. 1215-1226	Schreiber, S., et al., *OP34 VARSITY: a double-blind, double-dummy, randomised, controlled trial of vedolizumab versus adalimumab in patients with active ulcerative colitis.* Journal of Crohn's and Colitis, 2019. **13**(Supplement_1): p. S612-S613.
Sands 2022 (SEAVUE - maintenance)	Sands, Bruce E., et al. "Ustekinumab versus adalimumab for induction and maintenance therapy in biologic-naive patients with moderately to severely active Crohn's disease: a multicentre, randomised, double-blind, parallel-group, phase 3b trial." The Lancet 399.10342 (2022): 2200-2211.[135]	Sands, Bruce E., et al. "775d Ustekinumab versus adalimumab for induction and maintenance therapy in moderate-to-severe Crohn's disease: the SEAVUE study." Gastroenterology 161.2 (2021): e30-e31.[136]
Schreiber 2018	Schreiber, S., et al., *A phase 2, randomized, placebo-controlled study evaluating matrix*	Schreiber S, S.C., Friedenberg K, Seidler U, Bhandari BR ,Younes

	metalloproteinase-9 inhibitor, andecaliximab, in patients with moderately to severely active Crohn’s disease. Journal of Crohn's and Colitis, 2018. **12**(9): p. 1014-1020	Z, Wang K, Mckevitt M, Zhao S, Kanwar B, Sundy J, Lee SD, Loftus Jr EV, *ANDECALIXIMAB (ANTI-MMP9) INDUCTION THERAPY FOR CROHN’S DISEASE: A DOUBLE-BLIND, RANDOMIZED, PLACEBO-CONTROLLED, PHASE 2 STUDY.* United Eur Gastroenterol J, 2017. **5**: p. A302-3
Schwartz 2021 (ENTERPRISE)	Schwartz, D.A., et al., *Efficacy and safety of 2 vedolizumab intravenous regimens for perianal fistulizing Crohn’s disease: ENTERPRISE study.* Clinical Gastroenterology and Hepatology, 2022. **20**(5): p. 1059-1067. e9.	Schwartz, D.A., et al. *Efficacy and Safety of 2 Vedolizumab Iv Regimens in Patients with Perianal Fistulizing Crohn's Disease: Results of the Enterprise Study*. in *Gastroenterology*. 2020
Travis 2014 (CORE II)	Travis, S.P., et al., *Once-daily budesonide MMX in active, mild-to-moderate ulcerative colitis: results from the randomised CORE II study.* Gut, 2014. **63**(3): p. 433-441.	Sandborn, W.J., et al. *Budesonide-MMx® 9 mg for induction of remission of mild-to-moderate ulcerative colitis (UC): data from a multicenter, randomized, double-blind placebo-controlled study in the Europe, Russia, Israel and Australia*. in *Gastroenterology: Conference on Digestive Disease Week 2011: Abstract Supplement: Chicago, IL, May 07-10, 2011/AGA (American Gastroenterological Association) Institute. Philadelphia, PA: WB Saunders, 2011, vol. 140, iss. 5, suppl. 1, May*. 2011
Turner 2017	Turner, D., et al., *Once-versus twice-daily mesalazine to induce remission in paediatric ulcerative colitis: a randomised controlled trial.* Journal of Crohn's and Colitis, 2017. **11**(5): p. 527-533.	Turner, D., et al. *Once versus twice daily mesalazine to induce remission in paediatric ulcerative colitis: an investigator-initiated randomised controlled trial*. in *JOURNAL OF CROHNS & COLITIS*. 2016
VanAssche 2012 (SWITCH)	Van Assche, G., et al., *Switch to adalimumab in patients with Crohn's*	Van Assche, G.A., et al., *645 Switch to Adalimumab in*

	disease controlled by maintenance infliximab: prospective randomised SWITCH trial. Gut, 2012. **61**(2): p. 229-234.	*Patients With Crohn's Disease Controlled by Maintenance Infliximab. the Prospective Randomized Switch Study.* Gastroenterology, 2010. **138**(5): p. S-85.
Vaz 2019 (NEAT)	Vaz, K.K., et al., *Evaluation of a novel educational tool in adolescents with inflammatory bowel disease: the neat study.* Journal of pediatric gastroenterology and nutrition, 2019. **69**(5): p. 564	Vaz KH, Z.Y., Denson LA, Hommel KA, *Evaluation of a Novel Educational Tool in Adolescents With Inflammatory Bowel Disease: The NEAT Study.* Journal of pediatric gastroenterology and nutrition, 2016. **63**: p. S66.
Vermeire 2017 (FITZROY)	Vermeire, S., et al., *Clinical remission in patients with moderate-to-severe Crohn's disease treated with filgotinib (the FITZROY study): results from a phase 2, double-blind, randomised, placebo-controlled trial.* The Lancet, 2017. **389**(10066): p. 266-275.	Vermeire, S., et al., *812c Filgotinib (GLPG0634), an Oral JAK1 Selective Inhibitor, Induces Clinical Remission in Patients With Moderate-to-Severe Crohn's Disease: Results From the Phase 2 FITZROY Study Interim Analysis.* Gastroenterology, 2016. **150**(4): p. S1267.
Vogelaar 2013	Vogelaar, L., et al., *Fatigue management in patients with IBD: a randomised controlled trial.* Gut, 2014. **63**(6): p. 911-918.	Vogelaar, L., et al., *P503 Fatigue in IBD patients decreases with psychotherapy: results of a randomized controlled trial.* Journal of Crohn's and Colitis, 2013(7): p. S211-S212.
Volz 2016	Volz, M.S., A. Farmer, and B. Siegmund, *Reduction of chronic abdominal pain in patients with inflammatory bowel disease through transcranial direct current stimulation: a randomized controlled trial.* Pain, 2016. **157**(2): p. 429-437.	Pruess, M., A. Farmer, and B. Siegmund. *Inflammatory bowel disease-induced chronic abdominal pain can be ameliorated by transcranial direct current stimulation.* in *JOURNAL OF CROHNS & COLITIS*. 2016.
Watanabe 2012	Watanabe, M., et al., *Adalimumab for the induction and maintenance of clinical remission in Japanese patients with Crohn's disease.* Journal of Crohn's and Colitis, 2012. **6**(2): p. 160-173.	Hibi, T., et al., *Efficacy and Safety of Adalimumab for the Treatment of Japanese Patients with Moderately to Severely Active Crohn's Disease: Results from a Randomized Controlled Trial: 1061.* Official journal of the American College of Gastroenterology| ACG, 2008. **103**: p. S414-S415

Watanabe 2020 (induction)	Watanabe, K., et al., *Effects of vedolizumab in Japanese patients with Crohn's disease: a prospective, multicenter, randomized, placebo-controlled phase 3 trial with exploratory analyses.* Journal of gastroenterology, 2020. **55**: p. 291-306.	Ogata, H., et al., *Tu1746 – A Phase 3 Study of Vedolizumab for Induction and Maintenance Therapy in Japanese Patients with Moderate to Severe Crohn's Disease.* Gastroenterology, 2019. **156**(6): p. S-1109.
Watanabe 2020 (maintenance)	Watanabe, K., et al., *Effects of vedolizumab in Japanese patients with Crohn's disease: a prospective, multicenter, randomized, placebo-controlled phase 3 trial with exploratory analyses.* Journal of gastroenterology, 2020. **55**: p. 291-306.	Ogata, H., et al., *Tu1746 – A Phase 3 Study of Vedolizumab for Induction and Maintenance Therapy in Japanese Patients with Moderate to Severe Crohn's Disease.* Gastroenterology, 2019. **156**(6): p. S-1109.
Weizman 2019	Weizman, A.V., et al., *Providing Hospitalized Ulcerative Colitis Patients With Practice Guidelines Improves Patient-Reported Outcomes.* Journal of the Canadian Association of Gastroenterology, 2021. **4**(3): p. 131-136.	Weizman, A.V., et al., *Sa1863 – Patient Empowerment Through an Educational Intervention Improves Patient Satisfaction and Trust in Physician Among Hospitalized Patients with Ulcerative Colitis.* Gastroenterology, 2019. **156**(6): p. S-432-S-433.
Ye 2019	Ye, B.D., et al., *Efficacy and safety of biosimilar CT-P13 compared with originator infliximab in patients with active Crohn's disease: an international, randomised, double-blind, phase 3 non-inferiority study.* The Lancet, 2019. **393**(10182): p. 1699-1707.	Ye, B.D., et al., *814 - Phase Iii Randomized Controlled Trial to Compare Biosimilar Infliximab (CT-P13) with Innovator Infliximab in Patients with Active Crohn's Disease: 1-Year Maintenance and Switching Results.* Gastroenterology, 2018. **154**(6): p. S-167-S-168.

Appendix

EMBASE search (n=9605)

1 exp *Inflammatory Bowel Disease/ or (Inflammatory Bowel Disease* or IBD or Crohn* or Colitis or Enteritis or Proctocolitis or Colorectitis or Ileocolitis).ti,ab.
2 Randomized controlled trial/ or Controlled clinical study/ or randomization/ or intermethod comparison/ or double blind procedure/ or human experiment/ or (random$ or placebo or (open adj label) or ((double or single or doubly or singly) adj (blind or blinded or blindly)) or parallel group$1 or crossover or cross over or ((assign$ or match or matched or allocation) adj5 (alternate or group$1 or intervention$1 or patient$1 or subject$1 or participant$1)) or assigned or allocated or (controlled adj7 (study or design or trial)) or volunteer or volunteers).ti,ab. or (compare or compared or comparison or trial).ti. or ((evaluated or evaluate or evaluating or assessed or assess) and (compare or compared or comparing or comparison)).ti,ab.
3 (random$ adj sampl$ adj7 ("cross section$" or questionnaire$1 or survey$ or database$1)).ti,ab. not (comparative study/ or controlled study/ or randomi?ed controlled.ti,ab. or randomly assigned.ti,ab.)
4 Cross-sectional study/ not (randomized controlled trial/ or controlled clinical study/ or controlled study/ or (randomi?ed controlled or control group$1).ti,ab.)
5 (((case adj control$) and random$) not randomi?ed controlled).ti,ab.
6 (Systematic review not (trial or study)).ti.
7 (nonrandom$ not random$).ti,ab.
8 ("Random field$" or (random cluster adj3 sampl$)).ti,ab.
9 (review.ab. and review.pt.) not trial.ti.
10 "we searched".ab. and (review.ti. or review.pt.)
11 ("update review" or (databases adj4 searched)).ab.
12 (rat or rats or mouse or mice or swine or porcine or murine or sheep or lambs or pigs or piglets or rabbit or rabbits or cat or cats or dog or dogs or cattle or bovine or monkey or monkeys or trout or marmoset$1).ti. and animal experiment/
13 Animal experiment/ not (human experiment/ or human/)
14 or/3-13
15 2 not 14
16 and/1-2,15
17 limit 16 to (embase and yr="2010 - 2021")

MEDLINE (Ovid) search (n=9379)

1 exp Inflammatory Bowel Diseases/ or (Inflammatory Bowel Disease* or IBD or Crohn* or Colitis or Enteritis or Proctocolitis or Colorectitis or Ileocolitis).ti,ab.
2 ((randomized controlled trial or controlled clinical trial).pt. or (randomi?ed or placebo or randomly or trial or groups).ab. or drug therapy.fs.) not (exp animals/ not humans.sh.)
3 "and/1-2".m_titl.
4 limit 3 to yr="2010 - 2021"

CENTRAL (n=8919)

([mh "Inflammatory Bowel Diseases"] or (Inflammatory Bowel Disease* or IBD or Crohn* or Colitis or Enteritis or Proctocolitis or Colorectitis or Ileocolitis): with Publication Year from 2010 to 2021, in Trials
